# Does FXIII Deficiency Impair Wound Healing after Myocardial Infarction?

**DOI:** 10.1371/journal.pone.0000048

**Published:** 2006-12-20

**Authors:** Matthias Nahrendorf, Ralph Weissleder, Georg Ertl

**Affiliations:** 1 Medizinische Klinik und Poliklinik I, Universität Wrzburg Würzburg, Germany; 2 Center for Molecular Imaging Research, Massachusetts General Hospital, Harvard Medical School Charlestown, Massachusetts, United States of America; Baylor College of Medicine, United States of America

## Abstract

Inadequate healing of myocardial infarction may contribute to local expansion of the infarct, frequently leading to chamber dilation, heart failure, or myocardial rupture. Experimental evidence in mouse models suggests that Factor XIII might play a key role in wound healing, and low persistent values lead to increased incidence of cardiac rupture following myocardial infarction. Here we would like to share our initial clinical experiences with strikingly similar observations in patients with this grave disease, and compare these observations to experimental findings.

## Introduction

**Figure 1:**
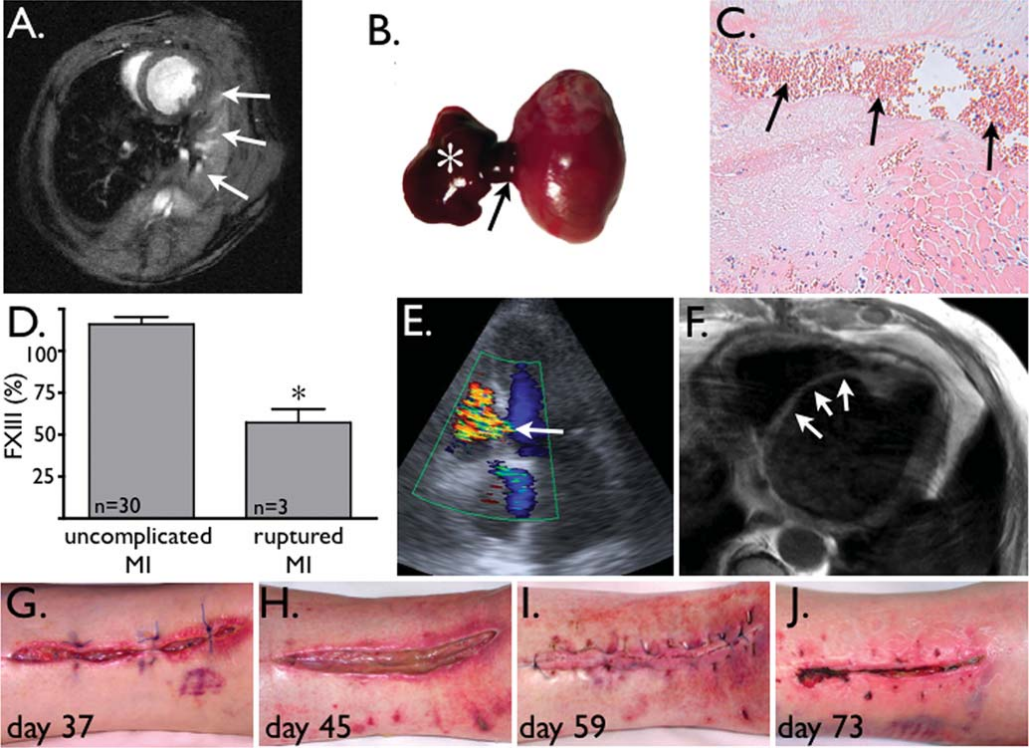
**A:** Short axis high resolution, high field cardiac MRI of a FXIII^−/−^ mouse 2 days after coronary ligation. Arrows: intrathoracic hematoma adjacent to experimental anterolateral infarction. **B:** Autopsy confirms a blood clot (asterisk) originating from myocardial rupture at the border zone (arrow) of the myocardial infarct. **C:** Histology of 1A shows rupture channel (arrows), filled with blood. **D:** In patients with ruptured MI, FXIII levels were significantly reduced (*p<0.01). **E:** Color Doppler echo of patient with new ventricular septum defect 7 days after myocardial infarction (arrow). **F:** MRI after VSD repair with patch (arrows). **G–I:** Explantation site of saphenous veins for CABG surgery displays delayed healing. **J:** 73 days after initial surgery, 3 revisions and 2 weeks after i.v. FXIII augmentation, the wound is closed.

Inadequate healing of myocardial infarction may contribute to local expansion of the infarct, frequently leading to chamber dilation, heart failure, or myocardial rupture [1]. Experimental evidence in mouse models suggests that Factor XIII might play a key role in wound healing and low persistent values lead to increased incidence of cardiac rupture following myocardial infarction. For example in heterozygous FXIII deficient mice (characterized by 50% plasma levels of FXIII) 100% die from cardiac rupture following MI (Figure 1 A–C) and FXIII replacement therapy reverts these findings [2]. Here we would like to share our initial clinical experiences with strikingly similar observations in patients with this grave disease.

## Results and Discussion

We investigated FXIII levels in three consecutive patients presenting with acute myocardial rupture following myocardial infarction. FXIII levels in these patients were only 57±8% of normal (Figure 1 D), whereas all other coagulation tests were normal or not consistently altered. One patient suffered a rupture of the ventricular septum 7 days following MI, presenting with sudden onset of cardiogenic shock and a new systolic heart murmur. The diagnosis was confirmed by Doppler (Figure 1 E). Cardiac catheterization showed a shunt volume of 66% and severe coronary artery disease. Therefore, surgical closure of the 2 cm VSD (Figure 1 F) was combined with coronary artery bypass grafting utilizing the patient’s saphenous veins. Six days later the patient presented again in cardiogenic shock, this time from a ruptured papillary muscle causing severe mitral valve insufficiency. The valve was replaced on the same day. Healing of the thoracotomy wound and lower extremity incisions was delayed for over 2 months (Figure 1 G–I), necessitating 3 surgical revisions. Repetitive swabs excluded infection, and the patient was not diabetic. During this time, FXIII levels were consistently reduced to 50%. Following the third revision, we initiated intravenous FXIII supplementation therapy (Fibrogammin 1,250 IE) and the skin wounds healed two weeks later (Figure 1 J). The other two patients died within 2 weeks after admission in cardiogenic shock.

To determine whether a truly causative relationship existed between FXIII activity and myocardial healing, we have recently studied myocardial repair in FXIII deficient mice [2]. All FXIII^−/−^ and FXIII^−/+^ mice died within 5 days after MI from left ventricular (LV) rupture and had locally reduced levels of FXIII as determined by imaging [2]. In contradistinction, FXIII^−/−^ that received 5 days of FXIII replacement therapy had normal survival rates, although cardiac MRI demonstrated worse LV remodeling in these reconstituted FXIII^−/−^. Likewise, skin healing was impaired in the same mouse model [3]. In conclusion, the reported clinical experiences and the parallel experimental data support the hypothesis that FXIII deficiency may impair healing in patients with MI while replacement therapy may alleviate complications. A prospective study is warranted to determine the clinical significance of these findings.
